# Prognostic Significance of DNAJB4 Expression in Gastric Cancer: Correlation with CD31, Caspase-3, and Tumor Progression

**DOI:** 10.3390/diagnostics15060652

**Published:** 2025-03-07

**Authors:** Chiao-Yin Cheng, Yen-Lin Chen, Hua Ho, Chun-Yen Huang, Sheng-En Chu, Yao-Jen Liang

**Affiliations:** 1 Graduate Institute of Applied Science and Engineering, Fu-Jen Catholic University, New Taipei 242062, Taiwan; 410068068@m365.fju.edu.tw (C.-Y.C.); femh93710@femh.org.tw (C.-Y.H.); 2 Department of Emergency Medicine, Far Eastern Memorial Hospital, New Taipei 220216, Taiwan; femh97657@femh.org.tw (H.H.); femh96839@femh.org.tw (S.-E.C.); 3 Department of Pathology, Tri-Service General Hospital, National Defense Medical Center, Taipei 114201, Taiwan; yenlinchen12742@mail.ndmctsgh.edu.tw; 4 Department of Emergency Medicine, National Taiwan University Hospital, Yun-Lin Branch, Douliu City 640203, Taiwan; 5 Department and Institute of Life Science, Fu-Jen Catholic University, New Taipei 242062, Taiwan

**Keywords:** DNAJB4, HLJ1, gastric cancer, CD31, caspase-3

## Abstract

**Background/Objectives:** Gastric cancer is one of the most common and lethal cancers worldwide, with particularly high incidence and mortality rates in East Asia and Europe. DNAJB4 has been shown to have prognostic implications in other cancer types; however, its expression patterns and role in gastric cancer have not been extensively studied. This study aimed to analyze DNAJB4 expression in gastric cancer and explore its association with clinical characteristics, molecular markers, and patient outcomes. **Methods:** We selected suitable tumor samples from 189 gastric cancer patients who had not undergone chemotherapy or radiotherapy, with 188 patients ultimately included in the analysis. Tissue microarray and immunohistochemistry were used to evaluate DNAJB4 expression, and the samples were divided into high- and low-expression groups based on the H-score. Multivariate logistic regression and survival analysis were conducted to identify influencing factors. **Results:** High DNAJB4 expression was significantly correlated with increased CD31 levels but was inversely associated with advanced cancer stages. Subgroup analysis revealed that in patients with advanced gastric cancer, high DNAJB4 expression was associated with increased caspase-3 levels and with elevated CD31 and decreased E-cadherin levels. **Conclusions:** High DNAJB4 expression was associated with both angiogenesis and apoptosis, indicating its complex role in gastric cancer progression. Although DNAJB4 promoted angiogenesis by increasing CD31 levels, it may also enhance apoptosis in tumor cells through caspase-3-induced apoptosis.

## 1. Introduction

Gastric cancer is one of the most common and fatal cancers, with particularly high incidence and mortality rates in East Asia and Europe. Non-modifiable risk factors include age, sex, and race, whereas modifiable risk factors include dietary and lifestyle factors, such as consumption of high-salt foods and foods containing *N*-nitroso compounds, smoking, alcohol consumption, and obesity [1,2,3,4,5,6]. The carcinogenesis of gastric cancer is multistage and involves various gene mutations and epigenetic alterations. Globally, the characteristics of gastric cancer vary by region, with the highest incidence in East Asia. In addition, tumors located in the gastric cardia are less frequent in Africa and Latin America [7,8].

The genes most frequently associated with gastric cancer in Asian populations include FAT4, a tumor suppressor whose mutations promote tumor growth and metastasis, and PIK3CA, an oncogene involved in the PI3K/AKT pathway. Other key genes include PCLO, KMT2C, PREX2, RNF213, RELN, PTPRT, and ZFHX3, which are associated with cancer progression, transcriptional regulation, and cell growth. Understanding these genetic alterations is critical for understanding the molecular characteristics of gastric cancer in Asians [2,8,9,10]. However, there are other genes that influence the occurrence and prognosis of gastric cancer. DnaJ heat shock protein (Hsp40) family member B4 (DNAJB4) is a member of the DnaJ protein family that functions as an auxiliary molecular chaperone for Hsp70 proteins. This gene is located on the human chromosome 1p31.1, primarily in the mitochondrial matrix. Although DNAJB4 shares homology with DNAJB1, it is less sensitive to heat shock and plays a housekeeping role, functioning under both stressful and normal cellular conditions. Unlike DNAJB1, which is involved in protein folding, DNAJB4 has limited effects on folding and aggregation inhibition. However, it binds to unfolded substrates and thus plays a role in the recognition of misfolded proteins [11,12,13].

The role of DNAJB4 has been studied in many cancer types. DNAJB4 is expressed at low levels in breast cancer tissues and cell lines, and its low expression is closely related to poor prognosis in patients with breast cancer [14]. Particularly, DNAJB4 is significantly downregulated in triple-negative breast cancer (TNBC) [15], and its low expression is associated with poor prognosis. DNAJB4 overexpression activates the Hippo signaling pathway, inhibits cancer cell proliferation and migration, and promotes TNBC cell apoptosis, suggesting its potential as a tumor suppressor and therapeutic target in breast cancer [15,16]. In non-small cell lung cancer (NSCLC), circ_0009043 is upregulated and suppresses DNAJB4 expression by sequestering miR-148a-3p, thereby promoting tumor progression [17]. DNAJB4 downregulation leads to malignant biological behavior in NSCLC cells, suggesting that DNAJB4 can be a potential anticancer target in NSCLC. In non-muscle-invasive bladder cancer (NMIBC), HERV-H-derived miR-4454 is upregulated, which inhibits the expression of the tumor suppressor genes DNAJB4 and SASH1, thereby promoting NMIBC progression. This suggests that miR-4454 plays a role in driving tumor growth [18].

In addition to breast, bladder, and non-small cell lung cancers, the role of DNAJB4 has also been reported in gastric cancer, although only in animal and cell experiments. Particularly, DNAJB4 plays a crucial tumor-suppressive role in gastric cancer. As a molecular chaperone, it directly interacts with E-cadherin (E-cad), regulating its stability and localization to the cell membrane. DNAJB4 promotes the stabilization of wild-type E-cad, enhances cell adhesion, and inhibits cancer cell invasiveness. When E-cad undergoes structural mutations, DNAJB4 recognizes these defects and directs the mutated E-cad to the proteasome for degradation, thereby reducing its half-life. Concurrent downregulation of DNAJB4 and E-cad is commonly observed in gastric cancer samples, highlighting their cooperative role in suppressing cancer progression and making DNAJB4 a potential therapeutic target [13,15,17]. Another study using a mouse model indicated that regulation by traditional Chinese medicine may influence DNAJB4 expression [19]. However, despite the importance of DNAJB4 expression in cancer, its specific role in gastric cancer has remained largely underexplored. Thus, this study aimed to analyze DNAJB4 expression in gastric cancer and investigate its correlation with prognosis.

## 2. Materials and Methods

### 2.1. Patients and Sample Collection

We selected 188 suitable gastric cancer tumor samples from patients treated after 2000. These tumors were primary lesions, and none of the patients underwent chemotherapy or radiotherapy. Patient information, including sex, age, tumor size, degree of differentiation, cancer stage, and survival status, was collected. Unfortunately, one tumor sample was insufficient for complete analysis and was excluded, thus leaving 188 patients in the final analysis.

### 2.2. Tissue Microarray Preparation and Immunohistochemistry

**Figure 1:**
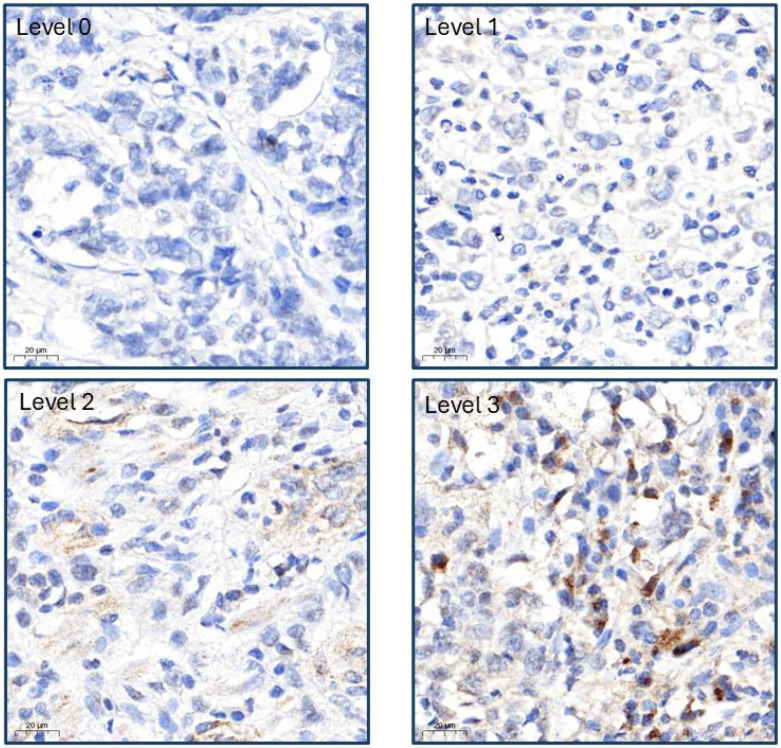
Differential staining intensities of DNAJB4 in tumor paraffin sections under immunohistochemistry (grades 0 to 3).

For each sample, we extracted a 0.2 cm diameter core and rearranged it to create a tissue microarray. The cores were then embedded in paraffin and cut into 0.5 µm thick sections, with each section mounted on slides for future immunohistochemical staining. Prestaining preparation involved heating the slides in a 65 °C oven for 1 h, followed by a rehydration process. The heated sections were placed in xylene for 10 min, placed in fresh xylene for another 10 min, and then immersed in absolute ethanol for 5 min. They were subsequently placed in 95% and 75% ethanol for 5 min each time and finally rinsed with distilled water for 10 min. The sections were then subjected to immunohistochemical staining using a Ventana BenchMark XT Automated Stainer (Ventana, Tucson, AZ, USA). We have selected several proteins that play critical roles in cancer. For apoptosis or inhibition of proliferation, the key proteins include DNAJB4 [20,21], caspase-3 [22], pAMPK [23], and HOXA5 [24]. Proteins involved in cell proliferation, growth promotion, or DNA synthesis are Ki67 [25], ERK [26], STAT3 [27], AXL [28], MCRS1 [29], and EphA5 [30]. Akt is identified as a key protein for the inhibition of autophagy [31], while CD31 is involved in angiogenesis [32]. For epithelial-to-mesenchymal transition (EMT), the relevant proteins are E-cadherin, *N*-cadherin, and Fibronectin [33]. HMGA1 plays a significant role in cancer metastasis, and IGF2BP1 is recognized for enhancing translation efficiency [34]. p53 plays a dual role in cancer by regulating both tumor suppression and immune response, where its loss or mutation enables immune evasion and cancer progression [35]. β-catenin plays a key role in cancer by driving tumor progression through the Wnt/β-catenin signaling pathway, influencing cell proliferation, metastasis [36]. Thereafter, they were washed with a buffer solution, followed by tissue retrieval with ethylenediaminetetraacetic acid for 40 min. To detect proteins, tissue samples were washed with a buffer solution and then retrieved using ethylenediaminetetraacetic acid (EDTA) for 40 min. The samples were incubated with antibodies targeting the aforementioned proteins at concentrations ranging from 1:25 to 1:500. The diluted antibodies were then applied to the tissue sections and incubated at 37 °C for 1 h. A DAB substrate chromogen solution was used for staining, and hematoxylin was used to counterstain the cell nuclei. The stained slides were evaluated and scored using the H-score system ranging from 0 to 300 (H-score = staining intensity (level 0–3, as shown in Figure 1) × percentage of staining) by Dr. Yen-Lin Chen at the Tri-Service General Hospital. We have also provided an image of negative immunostaining for comparison, as shown in Supplementary Figure S1.

### 2.3. Antibodies

Antibodies targeting key signaling and cellular markers include pAXL (R&D, AF2228, 1:100), pAkt (GeneTex, GTX11901, 1:50), pErk (R&D, AF1018, 1:200), pStat3 (Abcam, ab76315, 1:50), and pAMPK (Cell Signaling, 2535, 1:100). Markers related to cell proliferation and adhesion include Ki67 (BioLegend, 350503, 1:100), E-cadherin (Abcam, ab40772, 1:100), *N*-cadherin (Abcam, ab76011, 1:100), and Fibronectin (Santa Cruz, SC-8422, 1:50).

To study angiogenesis, CD31 (Abbiotec, 250590, 1:500) is used. Other cancer-related markers include Caspase3 (Cell Signaling, 9664, 1:100) for apoptosis, DNAJB4 (Novus, NBP1-81735, 1:25), HMGA1 (Cell Signaling, 12094, 1:50), HOXA5 (Abcam, ab82645, 1:50), IGF2BP1 (Abcam, ab82968, 1:75), and MCRS1 (Sigma, HPA039057, 1:400). Additionally, EhpA5 (Thermo, PA5-14581, 1:200) is included. p53 (Roche, 05278775001) and beta-Catenin (Roche, 05269016001) are ready-to-use antibodies, eliminating the need for dilution. These antibodies were carefully selected for their relevance in studying cellular signaling pathways, proliferation, adhesion, angiogenesis, and tumor progression in our research.

### 2.4. Statistical Analysis

We set an H-score cutoff of 1.84 for DNAJB4 and divided the samples into high- and low-expression groups. The normality of distribution of continuous variables was analyzed using the Kolmogorov–Smirnov test. As all continuous variables were non-normally distributed, statistical differences were analyzed using non-parametric tests. Categorical variables were expressed as counts and percentages, and statistical differences were analyzed using the chi-square test. Univariate logistic regression analysis was performed to identify variables significantly associated with DNAJB4 expression and outcomes. Significant variables in the univariate analysis, including sex, age, degree of differentiation, cancer stage (stages I–II and III–IV), and proteins, were then included in the multivariate logistic regression analysis to identify independent influencing factors. Risks were expressed as odds ratios (ORs) with their 95% confidence intervals (CIs). Finally, survival analysis was conducted using the Kaplan–Meier method. All statistical analyses were performed using SPSS version 26.0 (IBM Corp., Armonk, NY, USA).

## 3. Results

### 3.1. Patient Characteristics

Sex distribution, tumor size, and cancer stage were significantly different between the low (*n* = 61) and high (*n* = 127) DNAJB4 expression groups. Particularly, there were significant differences in the proportion of both female (78.7% vs. 62.2%) and male (21.3% vs. 37.8%) (*p* = 0.024) patients between the low- and high-expression groups. The median tumor size was significantly larger in the low-expression group (5.0 (3.9–7.0) vs. 4.1 (2.5–6.5), *p* = 0.042). Further, in terms of cancer stage, there were significantly fewer patients with early-stage disease (27.9% vs. 48.0%) and advanced-stage disease (72.1% vs. 52.0%) in the low-expression group (*p* = 0.009). In contrast, age and tumor differentiation (grade) were not significantly different between the groups. The patient characteristics are shown in Table 1.

### 3.2. Association of DNAJB4 Expression with Protein and Biomarker Levels

**Figure 2:**
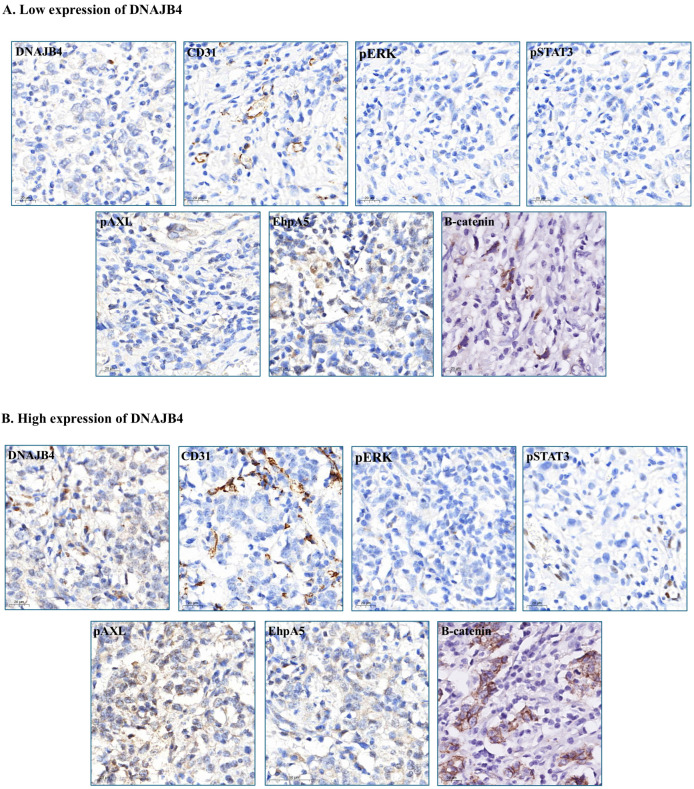
Immunohistochemistry of CD31, pERK, pSTAT3, pAXL, EhpA5, and β-catenin in the high and low DNAJB4 expression groups. (**A**) Low DNAJB4 expression. (**B**) High DNAJB4 expression. Scale bar, 20 μm for 400× magnification.

Significant differences were observed in several protein and biomarker levels between the low and high DNAJB4 expression groups. The between-group differences in the levels of caspase-3 (*p* = 0.051), E-cad (*p* = 0.052), and *N*-cadherin (*p* = 0.056) were close to statistical significance, suggesting the possibility of clear differences between the low- and high-expression groups. Particularly, CD31 (*p* < 0.001), pErk (*p* < 0.001), pStat3 (*p* = 0.001), pAXL (*p* = 0.046), and EhpA5 (*p* = 0.001) levels were significantly higher in the high-expression group, suggesting that high DNAJB4 expression was strongly associated with the expression of these proteins. In contrast, no significant between-group differences were found for the levels of Ki67 (*p* = 0.375), fibronectin (*p* = 0.671), pAkt (*p* = 0.279), pAMPK (*p* = 0.107), HMGA1 (*p* = 0.740), HOXA5 (*p* = 0.902), IGF2BP1 (*p* = 0.856), and MCRS1 (*p* = 0.295). These results reveal that the expression level of DNAJB4 affected the expression of certain proteins. Particularly, high DNAJB4 expression was associated with high levels of CD31, pErk, pStat3, pAXL, EhpA5, and β-catenin (Table 2, Figure 2). These findings provide important evidence for understanding the role of DNAJB4 expression in tumor biology.

### 3.3. Factors Associated with DNAJB4 Expression

In the univariate analysis, male sex was associated with a significantly higher risk than female sex (OR: 2.24, 95% CI: 1.10–4.56, *p* = 0.026), but this association became non-significant in the multivariate analysis (OR: 2.06, 95% CI: 0.91–4.66, *p* = 0.082). Age was also not significantly associated with outcomes in both the univariable (OR: 0.98, 95% CI: 0.95–1.00, *p* = 0.088) and multivariable (OR: 1.00, 95% CI: 0.97–1.03, *p* = 0.885) analyses. For tumor grade, with well-differentiated tumors as reference, neither moderately differentiated (multivariable OR: 2.75, 95% CI: 0.33–22.74, *p* = 0.348) nor poorly differentiated tumors (multivariable OR: 3.02, 95% CI: 0.36–25.59, *p* = 0.311) were significantly associated with outcomes. Tumor size showed a trend towards significance in the univariable analysis (OR: 0.92, 95% CI: 0.83–10.2, *p* = 0.095), but it was not significant in the multivariable analysis (OR: 0.96, 95% CI: 0.84–1.09, *p* = 0.494). With cancer stage I–II as reference, cancer stage III–IV showed a significant negative association with outcomes in both univariable (OR: 0.42, 95% CI: 0.22–0.81, *p* = 0.009) and multivariable (OR: 0.40, 95% CI: 0.17–0.97, *p* = 0.042) analyses, indicating a lower risk in advanced stages.

For biomarkers, CD31 levels were significantly associated with outcomes in both the univariable (OR 1.04, 95% CI 1.01–1.06, *p* = 0.002) and multivariable (OR 1.03, 95% CI 1.01–1.06, *p* = 0.015) analyses. pErk levels showed a significant association in the univariable analysis (OR: 1.14, 95% CI: 1.03–1.26, *p* = 0.015), but this significance was lost in the multivariable analysis (OR: 1.04, 95% CI: 0.95–1.15, *p* = 0.379). Similarly, pStat3 levels showed significance in the univariable analysis (OR: 1.97, 95% CI 1.10–3.55, *p* = 0.024) but became non-significant in the multivariable analysis (OR 1.33, 95% CI 0.84–2.11, *p* = 0.222). pAXL showed no significant trend in univariate analysis. (OR 1.08, 95% CI 0.99–1.18, *p* = 0.070). EhpA5 showed a significant association in the univariable analysis (OR 1.04, 95% CI 1.02–1.07, *p* = 0.002), but this association became non-significant in the multivariable analysis (OR 1.02, 95% CI 0.99–1.05, *p* = 0.203). β-catenin showed no significant trend in univariate analysis. (OR 1.00, 95% CI 0.99–1.00, *p* = 0.056) (Table 3).

These results indicate that although some factors, such as male sex, tumor size, and levels of certain biomarkers (i.e., CD31, pErk, pStat3, and EhpA5), were associated with outcomes, these factors have no independent influence. Notably, only cancer stage III–IV and CD31 expression were significantly and independently associated with outcomes. These findings suggest that advanced cancer stage and CD31 level are robust predictors of outcomes, whereas other factors have no significance (Table 3).

### 3.4. Survival Analysis

**Figure 3:**
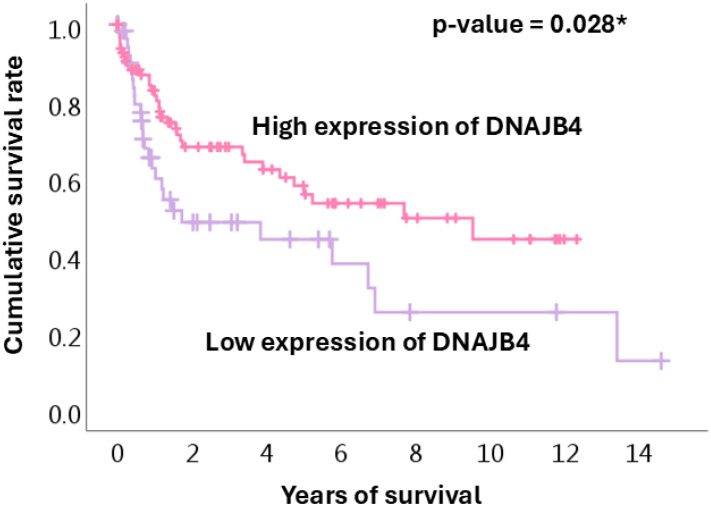
Kaplan–Meier analysis survival curves in the high and low DNAJB4 expression groups. Pink, high DNAJB4 expression group; purple, low DNAJB4 expression group. * *p* < 0.05.

The Kaplan–Meier curves of cumulative survival rates based on DNAJB4 expression levels are shown in Figure 3. Patients with high DNAJB4 expression consistently showed better survival rates throughout the 14-year follow-up period. Both groups showed a steep decline in survival during the first 2 years, but the decrease was more pronounced in the low-expression group. After 6 years, survival stabilized in the high-expression group, while that in the low-expression group continued to decline, resulting in significantly fewer long-term survivors. By the end of the observation period, the high-expression group exhibited considerably higher survival rates. This suggests that high DNAJB4 expression is associated with better long-term outcomes and may play a protective role against gastric cancer progression.

### 3.5. Role of DNAJB4 in Gastric Cancer by Stage

Several key differences emerged in the comparison between high and low DNAJB4 expression among patients with early- and late-stage cancers. Among the patients with early-stage cancer, males were significantly more likely to have high DNAJB4 expression than females (OR: 9.61, *p* = 0.042). Meanwhile, age, tumor differentiation, tumor size, and levels of biomarkers, such as *N*-cadherin and MCRS1, did not show significant associations (Table 4A). However, among the patients with late-stage cancer, high DNAJB4 expression was significantly associated with increased levels of caspase-3 (OR: 1.20, *p* = 0.013), CD31 (OR: 1.06, *p* = 0.004), and EphA5 (OR: 1.04, *p* = 0.033), but it was negatively correlated with E-cad expression (OR: 0.93, *p* = 0.011) (Table 4B). These findings suggest that in advanced gastric cancer stages, DNAJB4 plays a role in regulating apoptosis, angiogenesis, and cell adhesion. Meanwhile, its significance in early-stage cancer is more closely linked to the patient’s sex. Other variables, such as age, tumor differentiation, and tumor size, were not significantly associated with either disease stage.

## 4. Discussion

The current study found distinct differences between patients with gastric cancer with low and with high DNAJB4 expression. There were significant differences in DNAJB4 expression according to sex, tumor size, and cancer stage. Particularly, high DNAJB4 expression was more common in males and was associated with a smaller tumor size and an earlier cancer stage. The levels of biomarkers, such as CD31, pErk, pStat3, pAXL, and EhpA5 were significantly higher in the high DNAJB4 expression group. This suggests a strong association between DNAJB4 expression and these proteins, which may influence tumor behavior through pathways related to angiogenesis, apoptosis, and cell signaling. Although several factors, including male sex, tumor size, and certain biomarkers, showed significant associations with outcomes in the univariate analysis, only cancer stage and CD31 levels had a significant independent influence in the multivariate analysis, indicating their robust roles as predictors of patient outcomes. The results of the Kaplan–Meier survival analysis further supported the protective role of high DNAJB4 expression, with better long-term survival in patients with high DNAJB4 expression. These findings suggest that the DNAJB4 levels, particularly in conjunction with those of CD31, may serve as a valuable prognostic marker in gastric cancer, warranting further research on its biological role in cancer progression and patient outcomes.

Although there have been previous studies on DNAJB4 in gastric cancer models, these were only in animal and cellular models. Two studies have presented DNAJB4 as a potential target for cancer therapy because of its influence on protein folding and degradation, particularly its interaction with mutated proteins and its effects on cellular functions (e.g., adhesion) [13,19]. To our best knowledge, this study is the first to analyze the role of DNAJB4 in gastric cancer using human gastric cancer tumor paraffin sections for immunohistochemistry for common tumor markers and analyze them while also collecting relevant clinical data for more comprehensive human clinical research. The strength of our study lies in its multivariate analysis, the results of which emphasize that high CD31 levels and advanced cancer stage are strong predictors of outcomes and have an independent relationship with DNAJB4 expression. DNAJB4 may influence tumor biology, angiogenesis, and tumor progression.

A 2017 study by Acun et al. showed that DNAJB4 expression levels were reduced in breast cancer cells and correlated with poor prognosis, suggesting that incorporating DNAJB4 expression as a supplementary marker in the TNM classification system could enhance its accuracy, particularly in assessing metastatic risk and patient prognosis [20]. DNAJB4 plays a crucial role in tumor cell proliferation and migration, and its overexpression can inhibit tumor progression via the Hippo signaling pathway, indicating its potential as a molecular complement to the TNM system, especially for evaluating tumor aggressiveness and metastatic potential [16]. In the TNM classification, M represents distant metastasis, and low DNAJB4 expression is associated with high metastatic risk, highlighting its potential as a predictive biomarker. Furthermore, HERV-H-derived miR-4454 promotes the progression of NMIBC by downregulating tumor suppressor genes, such as DNAJB4, reinforcing the role of DNAJB4 as a tumor suppressor [18].

In colorectal cancer, miR-183 promotes malignant progression by inhibiting DNAJB4, underscoring the significance of miRNA-mediated regulation of tumor suppressor genes in cancer progression [37]. The miR-183/DNAJB4 axis may provide valuable molecular insights to complement TNM classification in colorectal cancer, improving the precision of tumor progression and metastasis assessments [37]. In addition to our finding that DNAJB4 has an independent impact on advanced-stage cancers, research on molecular biomarkers, such as DNAJB4, has revealed critical mechanisms in tumor progression, supporting their potential to supplement the TNM system towards a more individualized and accurate patient management. Incorporating such biomarkers into the TNM classification, particularly for evaluating metastatic risk and lymph node involvement, can significantly enhance treatment planning and prognostic evaluation. However, further investigation is needed to elucidate the molecular pathways that they regulate.

CD31 is an endothelial cell marker that plays a crucial role in tumor angiogenesis, particularly in gastric cancer [38,39,40,41,42]. A 2016 study by Tang et al. showed that CD31 expression was positively correlated galectin-1, which was overexpressed in cancer-associated fibroblasts of gastric cancer. Galectin-1 promotes angiogenesis by increasing CD31 and VEGF expression and enhancing endothelial cell proliferation, migration, and tube formation, thereby accelerating tumor growth. This suggests that CD31 is a valuable marker for assessing angiogenic activity in gastric cancer and is a potential target for anti-angiogenic therapy [43]. In a 2015 study by Blank et al., high CD31 levels were associated with increased tumor angiogenesis and poor treatment response [44], indicating its relevance as a prognostic marker and its potential for evaluating treatment efficacy, especially in anti-angiogenic therapies. Overall, these findings support that CD31 levels are not only a biomarker of tumor vascularization but also are a prognostic indicator that can improve individualized treatment for gastric cancer and other malignancies. In our study, high DNAJB4 expression increased CD31 expression but was negatively correlated with advanced-stage cancer. This result confirms that DNAJB4 affects CD31 expression.

The role of caspase-3 in cancer, particularly in gastric cancer, is complex and contradictory. As a critical executioner of apoptosis, caspase-3 is involved in the breakdown of cellular components during programmed cell death. In gastric cancer, high expression of caspase-3 expression is associated with improved prognosis, correlating with favorable features, such as smaller tumor size, earlier stages, and less lymph vascular involvement, suggesting a tumor-suppressive function [45]. Additionally, caspase-3 interacts with other proteins, such as caspase-8 and HLJ1, coordinating apoptosis and influencing tumor progression [46,47]. However, in advanced cancers, cleaved caspase-3 is associated with poor outcomes because it may promote tumor repopulation after apoptosis. This dual role highlights the capacity of caspase-3 to act as both a tumor suppressor and promoter depending on the cancer stage [48]. Enhancing caspase-3 activity can potentially strengthen apoptotic pathways, offering therapeutic benefits by suppressing tumor growth.

The current study showed that high DNAJB4 expression was a risk factor for increased CD31 levels but was negatively correlated with advanced-stage cancer. In an earlier study, CD31 was recognized as an important marker of angiogenesis, but this alone cannot explain the protective effect of high DNAJB4 expression in patients with gastric cancer. Therefore, we conducted a subgroup analysis of patients with advanced-stage gastric cancer and found significantly higher caspase-3 expression, along with significantly higher CD31 and lower E-cad levels in this subgroup (Table 4). Collectively, our results and those of previous studies support that high DNAJB4 expression promotes increased caspase-3 levels, thereby enhancing tumor cell apoptosis and providing a protective effect. However, the proliferation and apoptotic pathways in tumors are highly complex and require further confirmation.

In general, our results support that high DNAJB4 expression in gastric cancer is associated with increased levels of CD31, a key marker of angiogenesis, but is negatively correlated with advanced-stage cancer. Although CD31 is generally linked to tumor progression, the protective role of DNAJB4 remains unclear. Subgroup analysis of patients with advanced gastric cancer showed that high DNAJB4 expression was associated with elevated levels of caspase-3, which promotes apoptosis, as well as increased CD31 and decreased E-cad levels, both of which contribute to poor tumor outcomes. This suggests that DNAJB4 may exert its protective effect by enhancing caspase-3-mediated apoptosis, despite the complexity of tumor proliferation and apoptosis pathways. Further investigations are needed to fully understand the mechanisms underlying these interactions and their impact on tumor behavior.

This study had some limitations. First, the sample size, although reasonable for 188 patients with gastric cancer, may still be insufficient to capture the full range of genetic and protein expression variabilities observed in larger, more diverse populations. Additionally, all tumor samples were collected from a single institution, which may have introduced selection bias and limited the generalizability of the findings to broader populations or different geographic regions. Second, although associations were observed between DNAJB4 expression and various clinical and molecular markers, this was a correlation-based study, and causality could not be definitively established. Third, although DNAJB4 expression was analyzed and its relationship with other proteins and patient outcomes was investigated, the specific molecular mechanisms by which DNAJB4 affected cancer progression, angiogenesis, and apoptosis remained unclear, thus requiring further functional studies. Fourth, the reliance on immunohistochemical techniques to assess protein expression may introduce variability, and the use of automated staining may not fully capture subtle expression differences. Finally, although multivariate analysis was performed to control for potential confounding factors, there may still be unaccounted confounders affecting the observed relationships (e.g., other genetic and environmental factors) that were not included in this analysis. Further studies, including larger multicenter cohorts and mechanistic investigations, are needed to validate the findings and clarify the role of DNAJB4 in gastric cancer.

## 5. Conclusions

High DNAJB4 expression in gastric cancer is associated with increased CD31 expression, a key marker of angiogenesis, but is inversely associated with advanced cancer stage. Although CD31 typically indicates tumor progression, the protective role of DNAJB4 appears related to caspase-3-mediated apoptosis, as shown in the late-stage subgroup analysis. The complex interplay between proliferation and apoptosis in tumors suggests that further research is needed to clarify the impact of DNAJB4 on tumor behavior.

## Figures and Tables

**Table 1:** Patient characteristics.

Characteristic	Low DNAJB4 Expression Group	High DNAJB4 Expression	Total	*p*-Value
Sex				0.024 *
Female	48 (78.7%)	79 (62.2%)	127 (67.6%)	
Male	13 (21.3%)	48 (37.8%)	61 (32.4%)	
Age (years)	75.0 (69.0–80.0)	74.0 (60.0–80.0)	75.0 (63.0–80.0)	0.299
Differentiation (grade)				0.911
Well	2 (3.3%)	5 (4.0%)	7 (3.8%)	
Moderate	16 (26.7%)	30 (24.0%)	46 (24.9%)	
Poor	42 (31.8%)	90 (72.0%)	132 (71.4%)	
Tumor size (cm)	5.0 (3.9–7.0)	4.1 (2.5–6.5)	4.5 (2.9–7.0)	0.042 *
Cancer stage				0.009 **
I–II	17 (27.9%)	61 (48.0%)	78 (41.5%)	
III–IV	44 (72.1%)	66 (52.0%)	110 (58.5%)	

Data are presented as *n* (%) or the median (Q1–Q3 range). * *p* < 0.05, ** *p* < 0.01.

**Table 2:** H-scores for various cancer biomarkers.

Biomarker	Low DNAJB4 Expression Group	High DNAJB4 Expression Group	Total	*p*-Value
Caspase-3	4.2 (2.8–5.5)	4.7 (3.2–7.4)	4.4 (3.2–6.6)	0.051
Ki67	14.8 (4.0–41.3)	11.9 (3.4–32.7)	13.61 (3.5–36.0)	0.375
CD31	15.7 (9.3–24.0)	22.8 (14.8–37.1)	21.2 (13.1–34.4)	<0.001 ***
E-cad	106.9 (102.2–118.2)	104.8 (101.4–111.0)	105.6 (101.8–112.7)	0.052
*N*-cad	5.5 (2.3–13.1)	7.6 (3.7–11.5)	6.9 (3.0–11.8)	0.056
Fibronectin	36.7 (7.7–97.0)	38.9 (2.5–104.1)	37.7 (3.6–101.1)	0.671
pAkt	10.0 (3.8–17.8)	10.3 (3.7–30.4)	10.1 (3.8–26.4)	0.279
pErk	0.3 (0.1–1.1)	1.6 (0.4–5.8)	0.9 (0.2–3.3)	<0.001 ***
pStat3	0.1 (0.0–0.3)	0.3 (0.0–1.6)	0.2 (0.0–0.7)	0.001 **
pAMPK	4.1 (2.9–7.7)	3.8 (2.6–5.6)	3.9 (2.6–6.1)	0.107
pAXL	1.7 (1.1–4.1)	2.2 (1.5–4.4)	2.2 (1.3–4.3)	0.046 *
HMGA1	6.4 (0.7–62.4)	6.9 (1.0–29.0)	6.7 (1.0–44.9)	0.740
HOXA5	33.7 (8.9–77.8)	34.8 (12.1–57.0)	34.2 (11.2–64.2)	0.902
IGF2BP1	43.8 (14.3–64.1)	42.0 (19.2–61.4)	43.0 (19.1–63.6)	0.856
MCRS1	109.7 (71.3–165.9)	102.8 (69.3–143.9)	103.5 (70.5–146.9)	0.295
EhpA5	5.2 (2.7–9.6)	10.3 (3.4–28.2)	7.7 (3.2–19.8)	0.001 **
p53	1.6 (0.0–14.6)	2.1 (0.4–11.9)	2.1 (0.3–12.0)	0.686
β-catenin	154.0 (77.4–194.8)	122.3 (29.8–177.2)	134.2 (37.7–187.2)	0.027 *

Data are presented as the median (Q1–Q3). * *p* < 0.05, ** *p* < 0.01, *** *p* < 0.001.

**Table 3:** Logistic regression analyses of the factors associated with gastric cancer outcomes.

Characteristic	Univariable	*p*-Value	Multivariable	*p*-Value
Sex				
Female	Reference		Reference	
Male	2.24 (1.10, 4.56)	0.026 *	2.06 (0.91–4.66)	0.082
Age	0.98 (0.95–1.00)	0.088	1.00 (0.97–1.03)	0.885
Differentiation (grade)				
Well	Reference		Reference	
Moderate	0.75 (0.13–4.31)	0.747	2.75 (0.33–22.74)	0.348
Poor	0.86 (0.16–4.60)	0.857	3.02 (0.36–25.59)	0.311
Tumor size	0.92 (0.83–10.2)	0.095	0.96 (0.84–1.09)	0.494
Cancer stage				
I and II	Reference		Reference	
III and IV	0.42 (0.22–0.81)	0.009 **	0.40 (0.17–0.97)	0.042 *
Marker				
CD31	1.04 (1.01–1.06)	0.002 **	1.03 (1.01–1.06)	0.015 *
pErk	1.14 (1.03–1.26)	0.015 *	1.04 (0.95–1.15)	0.379
pStat3	1.97 (1.10–3.55)	0.024 *	1.33 (0.84–2.11)	0.222
pAXL	1.08 (0.99–1.18)	0.070		
EhpA5	1.04 (1.02–1.07)	0.002 **	1.02 (0.99–1.05)	0.203
β-catenin	1.00 (0.99–1.00)	0.056		

* *p* < 0.05, ** *p* < 0.01.

**Table 4:** (**A**) Multiple logistic regression analysis in patients with stage I–II gastric cancer. (**B**) Multiple logistic regression analysis in patients with stage III–IV cancer.

(**A**)		
**Characteristic**	**Multivariable**	***p*-Value**
Sex		
Female	Reference	
Male	9.61 (1.09–85.09)	0.042 *
Age	1.01 (0.95–1.07)	0.742
Differentiation		
Well	Reference	
Moderate	2.28 (0.22–23.16)	0.486
Poor	3.80 (0.32–44.89)	0.289
Tumor size	0.79 (0.57–1.10)	0.164
Biomarker		
*N*-cad	1.01 (0.99–1.04)	0.389
MCRS1	0.99 (0.97–1.00)	0.141
(**B**)		
**Characteristic**	**Multivariable**	***p*-Value**
Sex		
Female	Reference	
Male	1.16 (0.40–3.37)	0.788
Age	0.97 (0.93–1.01)	0.518
Differentiation (grade)		
Well	Reference	
Moderate	0.67 (0.16–2.81)	0.583
Poor	N/A	N/A
Tumor size	0.93 (0.78–1.10)	0.397
Biomarker		
Caspase-3	1.20 (1.04–1.39)	0.013 *
CD31	1.06 (1.02–1.10)	0.004 **
E-cad	0.93 (0.87–0.98)	0.011 *
EhpA5	1.04 (1.00–1.07)	0.033 *

* *p* < 0.05, ** *p* < 0.01.

## Data Availability

The data presented in this study are available on request from the corresponding author.

## Supplementary Materials

The following supporting information can be downloaded at: https://www.mdpi.com/article/10.3390/diagnostics15060652/s1, Figure S1: The negative control for DNAJB4 was applied under the same staining conditions as the study samples.
