# Supplementary material for: Prognostic Significance of DNAJB4 Expression in Gastric Cancer: Correlation with CD31, Caspase-3, and Tumor Progression
Source: Diagnostics (Basel). 2025 Mar 7;15(6):652. doi: 10.3390/diagnostics15060652 (PMC11941126; doi:10.3390/diagnostics15060652)
Supplement: Supplementary file 1 [file diagnostics-15-00652-s001.zip › diagnostics-3264897-supplementary.pdf]

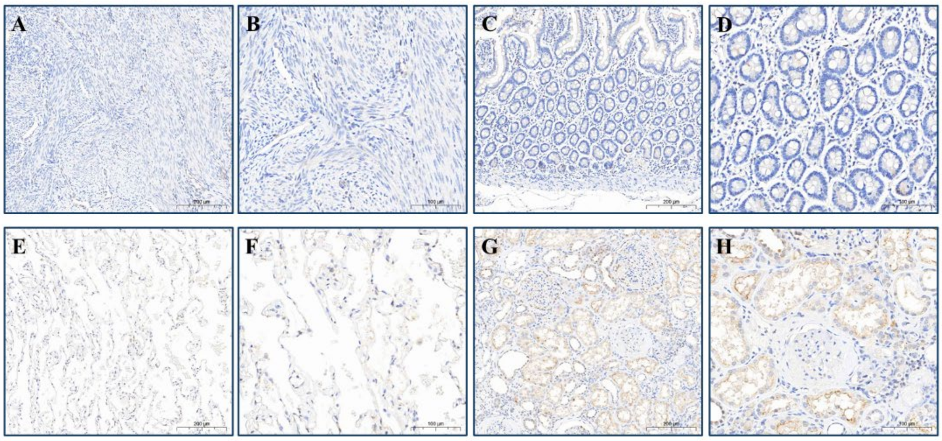

Supplementary Figure S1. The negative control for DNAJB4 was applied under the same staining conditions as the study samples. Figures A and B depict the myometrium of the uterus. Figures C and D show the small intestine. Figures E and F illustrate lung tissue, where some macrophages display mild immunostaining. Figures G and H represent the kidney cortex, where mild to moderate staining intensity is observed in the proximal tubule epithelium, indicating non-specific staining in the immunohistochemical assay.
